# Thoracic mesenchymal malignant tumors and programed cell death ligand‐1 status: Clinicopathologic and prognostic analysis of eight pulmonary sarcomatoid carcinomas and eight malignant mesotheliomas

**DOI:** 10.1111/1759-7714.14177

**Published:** 2021-10-15

**Authors:** Kanji Otsubo, Hiroki Sakai, Hiroyuki Kimura, Tomoyuki Miyazawa, Hideki Marushima, Koji Kojima, Naoki Furuya, Masamichi Mineshita, Motohiro Chosokabe, Junki Koike, Hisashi Saji

**Affiliations:** ^1^ Departments of Chest Surgery St. Marianna University School of Medicine Kawasaki Japan; ^2^ Division of Respiratory Medicine, Department of Internal Medicine St. Marianna University School of Medicine Kawasaki Japan; ^3^ Department of Pathology St. Marianna University School of Medicine Kawasaki Japan

**Keywords:** immune checkpoint inhibitor, malignant pulmonary methotelioma, programed cell death ligand‐1, pulmonary sarcomatoid carcinoma

## Abstract

**Background:**

The current study aimed to evaluate the significance of clinicopathological factors, particularly the immunohistochemistry of programed cell death ligand‐1 (PD‐L1), in eight cases each of pulmonary sarcomatoid carcinoma (PSC) and malignant pleural mesothelioma (MPM) at our hospital.

**Methods:**

From January 2004 to December 2020, a total of 16 consecutive patients (eight with PSC and eight with MPM diagnosed via surgical resection or biopsy) were included in this study. After retrospectively reviewing the patient characteristics, the associations between PD‐L1 status and age, sex, stage, histological type, and prognosis were investigated.

**Results:**

PD‐L1‐positive staining was observed in four (50%) PSC cases and one (12.5%) MPM case. Among the four PD‐L1‐positive PSC cases, two showed high PD‐L1 expression in the vimentin‐positive sarcomatoid compartment. Moreover, among those with PSC, two survived for about 10 years, whereas the others died within 5 years. No clear correlation was found between PD‐L1 expression and prognosis. Among the patients with MPM, four survived for more than 2 years, with the longest being 9 years. Among MPM cases who received nivolumab, one patient with positive PD‐L1 staining in the sarcomatoid survived, whereas the other with negative PD‐L1 staining did not.

**Conclusion:**

The present study showed that sarcomatoid carcinoma had a higher PD‐L1 expression compared to non‐small‐cell lung cancer and that both PSC and MPM tended to exhibit PD‐L1 positivity in the sarcomatoid compartment. Moreover, while immune checkpoint inhibitors may somewhat prolong the prognosis of both tumors, further studies with a larger cohort are necessary to confirm our results.

## Graphical abstract

In malignant pleural mesothelioma (MPM) and pulmonary sarcomatoid carcinoma (PSC), which are thoracic mesenchymal malignant tumors, programed cell death ligand‐1 (PD‐L1) was positive in the vimentin‐positive part or the sarcomatoid area, found to be clinicopathologically significant. In MPM, the effectiveness of immune checkpoint inhibitors (ICIs) has been shown, especially in nonepithelioid type. Our results support the possibility that ICIs will work as well as MPM if PSC is also positive for PD‐L1 and has mesenchymal features such as vimentin‐positive or sarcomatoid compartments.

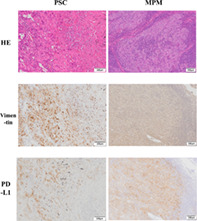

## INTRODUCTION

Pulmonary sarcomatoid carcinoma (PSC) and malignant pulmonary mesothelioma (MPM) are two uncommon histologic subtypes of thoracic malignant tumors that exhibit a mesenchymal pattern and have poor prognosis. In fact, studies have shown that the PSC and MPM have an overall 5‐year survival rate of approximately 20% and 10%, respectively.^1^, ^2^, ^3^ Although conventional platinum‐based chemotherapies have been known to have poor effects in both malignant tumors, adequate medications and regimens have yet to be established.

PSC is a rare histologic subtype of non‐small‐cell lung cancer (NSCLC), accounting for approximately 0.3–1.3% of all lung malignancies. This histologic subtype mostly occurs in male smokers during their sixth and seventh decades. The World Health Organization (WHO) defines PSC as a poorly differentiated NSCLC that contains a sarcoma or sarcomatoid component.^4^, ^5^, ^6^, ^7^ PSC can be histologically classified into five types, namely pleomorphic carcinoma, spindle‐cell carcinoma, giant cell carcinoma, carcinosarcoma, and pulmonary blastoma, with pleomorphic, spindle‐cell, and giant cell carcinoma accounting for most of such subtypes. The poor prognosis of PSC has been attributed to the ineffectiveness of first‐line platinum‐based chemotherapy.^8^, ^9^ Moreover, its nonspecific symptoms have often resulted in late‐stage diagnosis, with some patients presenting with no indications for surgical resection. Reports have shown that two‐thirds of patients with PSC have disease progression on first evaluation, with a mean median survival of 11.0–13.3 months and a 5‐year overall survival of approximately 20%.^3^, ^7^

MPM is another rare yet aggressive malignant tumor originating from the pleura that has been associated with asbestos exposure.^1^, ^2^, ^10^ MPM can be histologically classified into three types, namely epithelioid, sarcomatoid, and biphasic.^11^ Notably, evidence has shown that the nonepithelioid type has worse prognosis compared to the epithelioid type. The only approved drug therapy for MPM has been a combination of platinum and a folate antimetabolite, such as pemetrexed. Although bevacizumab, a targeted drug, has been added to the regimen, its usage varies by region.^12^ Nonetheless, long‐term survival with chemotherapy remains poor. Recently, immune checkpoint inhibitors (ICIs), such as programed cell death ligand‐1 (PD‐L1) inhibitors, have dramatically improved the prognosis of lung carcinoma.^13^, ^14^ Several reports examining PD‐L1 positivity in PSC have suggested that the presence or absence of PD‐L1 determined via immunohistochemistry can predict the effects of PD‐L1 inhibitors.^15^, ^16^, ^17^ According to the aforementioned studies, PSC has higher PD‐L1 positivity rates compared to conventional NSCLC, suggesting that PD‐L1 inhibitors may be an optimal treatment for PSC. Meanwhile, clinical trials on MPM have indicated the therapeutic effect of a combination therapy comprising anti‐PD‐L1 antibody and anti‐cytotoxic T‐lymphocyte associated antigen (CTLA) antibody. However, several studies have reported that the PD‐L1 positivity rate does not aid in the prediction of the therapeutic effect of this combination therapy.^18^, ^19^, ^20^ The current study therefore aimed to evaluate the significance of the clinicopathological factors, including the immunohistochemistry of PD‐L1, in eight cases each of PSC and MPM at our hospital.

## METHODS

### Patients

This study included consecutive patients with PSC or MPM diagnosed via surgical resection or biopsy at St. Marianna University School of Medicine Hospital from January 2004 to December 2020. The follow‐up period lasted as long as medical records existed, with the maximum duration being 10 years. The histopathological diagnosis was confirmed by two pathologists (JK and MC) according to the current 2015 WHO classification of lung tumor. The clinical characteristics, surgical staging, tumor‐node‐metastasis staging (8th edition), treatments, and outcomes were collected from the medical records. This study was approved by the institutional review board of the St. Marianna University School of Medicine in Kanagawa, Japan (Accession No. 1461). Prior to study initiation, written informed consent was obtained from all participants. All patient data remained confidential throughout this research.

### Immunohistochemistry

To measure PD‐L1 expression, this study used a murine anti‐PD‐L1 monoclonal antibody (E1L3N; Cell Signaling). Briefly, serial 3‐μm thick tissue sections were cut from formalin‐fixed, paraffin‐embedded blocks. Antigen retrieval was performed in a 97°C water bath for 15 min in pH 9 solution. Intrinsic peroxidase activity was blocked using hydrogen peroxide for 10 min. After washing the section with a phosphate buffer solution, automated immunohistochemistry was conducted (Histostainer 48A; Nichirei Bioscience Inc.). The PD‐L1 antibody dilution was 1:400. PD‐L1 expression was calculated as the percentage of complete or partial membrane staining in a section that included at least 100 viable tumor cells. Positive and negative expressions of PD‐L1 were defined as >1% and <1% staining of the tumor cell membrane, respectively.

To measure vimentin expression, we used a murine antivimentin monoclonal antibody (clone V9 Link; Dako). Briefly, serial 3‐μm thick tissue sections were cut from formalin‐fixed, paraffin‐embedded blocks, deparaffinized in xylene, and rehydrated through a graded series of ethanol concentrations. Antigen retrieval was performed in a 97°C water bath for 15 min in pH 9 solution. Intrinsic peroxidase activity was blocked using hydrogen peroxide for 5 min. After washing the section with a phosphate buffer solution, automated immunohistochemistry was conducted (Histostainer 48A; Nichirei Bioscience Inc). The vimentin antibody dilution was 1:2. Positive and negative vimentin expressions were defined as >50% and <1% staining of tumor cell membrane, respectively.

## RESULTS

### Patient characteristics

A total of eight consecutive patients were pathologically diagnosed with PSC based on surgical tissue or biopsy samples (Table 1). The median age at diagnosis was 72.5 years old. Among such patients, seven were male (87.5%), and three, one, one, and three had stage I (37.5%), stage II (12.5%), stage III (12.5%), and stage IV (37.5%) disease, respectively. Moreover, three (37.5%) were smokers, two were never smokers, and the rest had unknown smoking history. Pleomorphic carcinoma (50%) was the most frequently identified subtype, with one case having spindle‐cell carcinoma and another having giant cell carcinoma. The other tumors were unclassifiable.

**TABLE 1:** Main clinicopathological characteristics of PSCs

Age	Sex	Stage	Smoking	Treatment	Histology	PD‐L1	Survival	Outcome
70	M	IA	No	Surgery	Sarcomatoid carcinoma	Negative	Alive	Multiple bone metastasis 9 months after surgery
76	F	IA	No	Surgery	Pleomorphic carcinoma	Positive	Alive	Alive 10 years after surgery
61	M	IA	Unknown	Surgery	Spindle‐cell carcinoma	Positive	Alive	Alive 9 years after surgery
66	M	IIB	Unknown	Chemotherapy	Pleomorphic carcinoma	Negative	Death	Dead 2 months after diagnosis
75	M	IIIA	Yes	Surgery	Pleomorphic carcinoma	Positive	Death	Multiple bone metastasis 3 months after surgery
66	M	IV	Unknown	Radiation	Sarcomatoid carcinoma	Negative	Death	Unknown
90	M	IV	Yes	Radiation	Pleomorphic carcinoma	Positive	Death	Dead 3 months after diagnosis
79	M	IV	Yes	BSC	Giant cell carcinoma	Negative	Death	Dead 2 months after diagnosis

*Abbreviations*: BSC, best supportive care; F, female; M, male.

A total of eight consecutive patients were pathologically diagnosed with MPM based on surgical tissue or biopsy samples (Table 2). The median age at diagnosis was 66.5 years old. Among such patients, seven (87.5%) were male, and seven and one presented with stage I (87.5%) and stage III (12.5%) disease at the time of diagnosis, respectively. Seven cases were identified as epithelial type, whereas the remaining were sarcomatoid type.

**TABLE 2:** Main clinicopathological characteristics of MPMs

Age	Sex	Stage	Surgery	Chemotherapy	Histology	PD‐L1 status	Survival	Outcome
71	M	IA	Intrathoracic debridement	First: CDDP plus PEM 2 cources Second: nivolumab (PD)	Epithelioid type	Negative (Figure 2a,b)	Alive (4 years)	Best supportive care 3 years after surgery
71	M	IA	Biopsy	First: CDDP plus PEM 2 cources Second: nivolumab (PR)	Epithelioid type	Positive (Figure 2c,d)	Alive (2 years)	
53	F	IA	Total pleural pneumonectomy	CDDP plus PEM 2 cources	Epithelioid type	Negative	Alive (9 years)	
57	M	IA	Total pleural pneumonectomy	CDDP plus PEM 2 courses (NAC)	Epithelioid type	Negative	Alive (7 years)	Recurrence 6 years after surgery followed by PEM
62	M	IB	Total pleural pneumonectomy	CDDP plus PEM 2 cources	Epithelioid type	Negative	Death (1 year)	Respiratory failure due to pleural effusion
78	M	IB	Biopsy	CDDP plus PEM	Biphasic type	Negative	Death (3 months)	Respiratory faille due to pleural effusion
71	M	IB	Total pleural pneumonectomy	CDDP plus PEM 2 cources	Epithelioid type	Negative	Death (2 months)	Dead 2 months after surgery
62	M	IIIA	Total pleural pneumonectomy		Epithelioid type	Negative	Death (5 months)	Respiratory failure due to pleural effusion

Abbreviations: CDDP, cisplatin; F, female; M, male; NAC, neoadjuvant chemotherapy; PD, partial disease; PEM, pemetrexed sodium hydrate; PR, partial response.

### Pathological findings and PD‐L1 status

**FIGURE 1:**
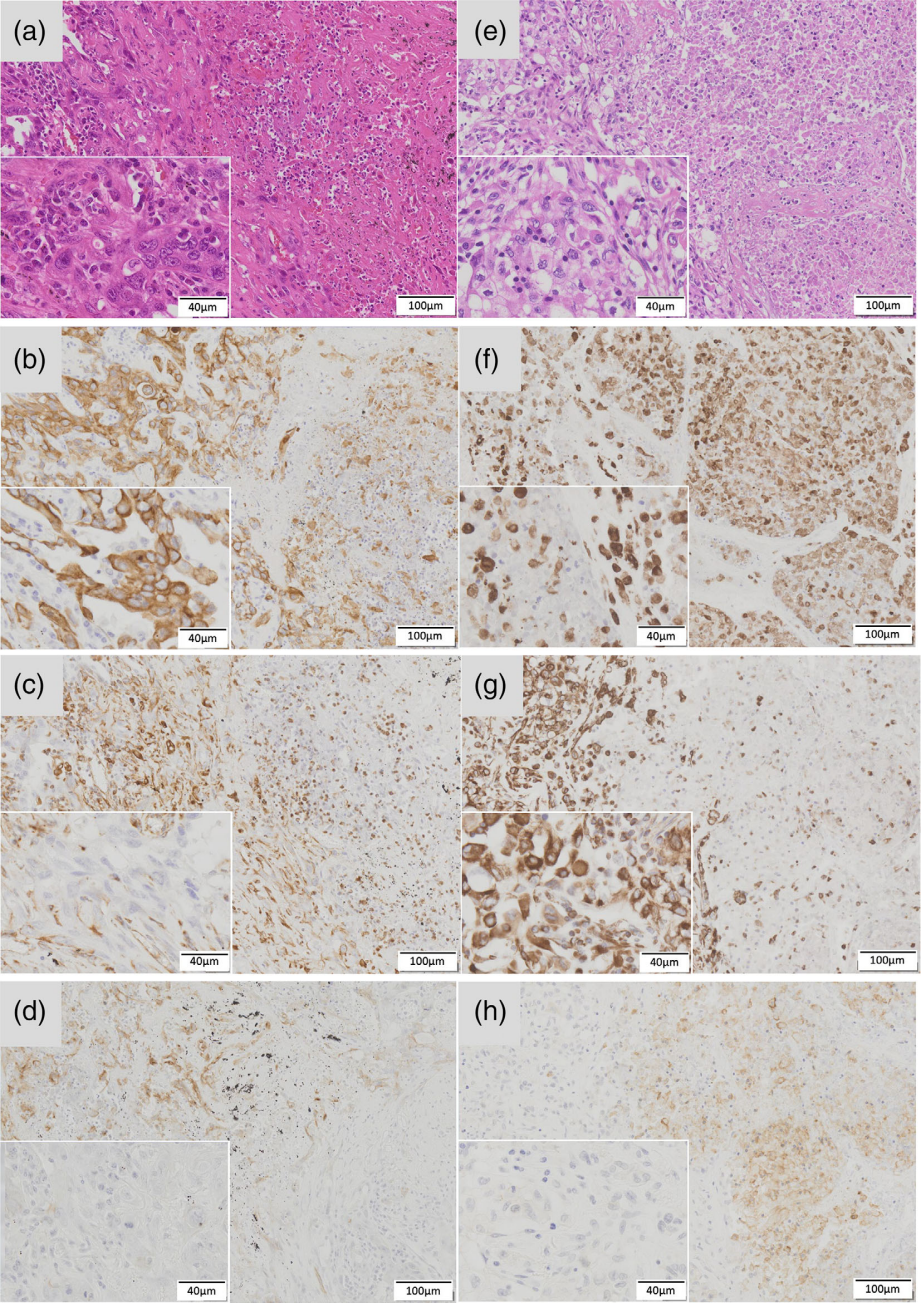
Immunohistochemistry of pulmonary sacomatoid carcinoma. (a)–(d) and (e)–(h) are the same specimen. (a) and (e) show hematoxylin–eosin staining, (b) and (f) show cytokeratin AE1/AE3 immunohistochemistry, (c) and (g) show vimentin immunohistochemistry, and (d) and (f) show programed cell death ligand‐1 (PD‐L1) immunohistochemistry. (a)–(d) show PD‐L1 expression in the vimentin‐positive sarcomatoid area, while (e)–(h) show PD‐L1 expression in the vimentin‐negative epithelioid area. The lower left quarter of each image is an enlarged image of the PD‐L1 negative part, which is composed of atypical cells with a high nuclear/cell ratio and different sizes, indicating that it is tumor

Among cases with PSC, four (50%) had PD‐L1‐positive staining in the tumor cells. Among these four cases, two showed high PD‐L1 expression. Moreover, three cases, two of which had high PD‐L1 expression, were PD‐L1‐positive only in the vimentin‐positive sarcomatoid compartment, whereas the remaining case was PD‐L1‐positive in the epithelial areas (Figure 1). A comparison with reported studies on PD‐L1 expression in PSC is provided in Table 2.

**FIGURE 2:**
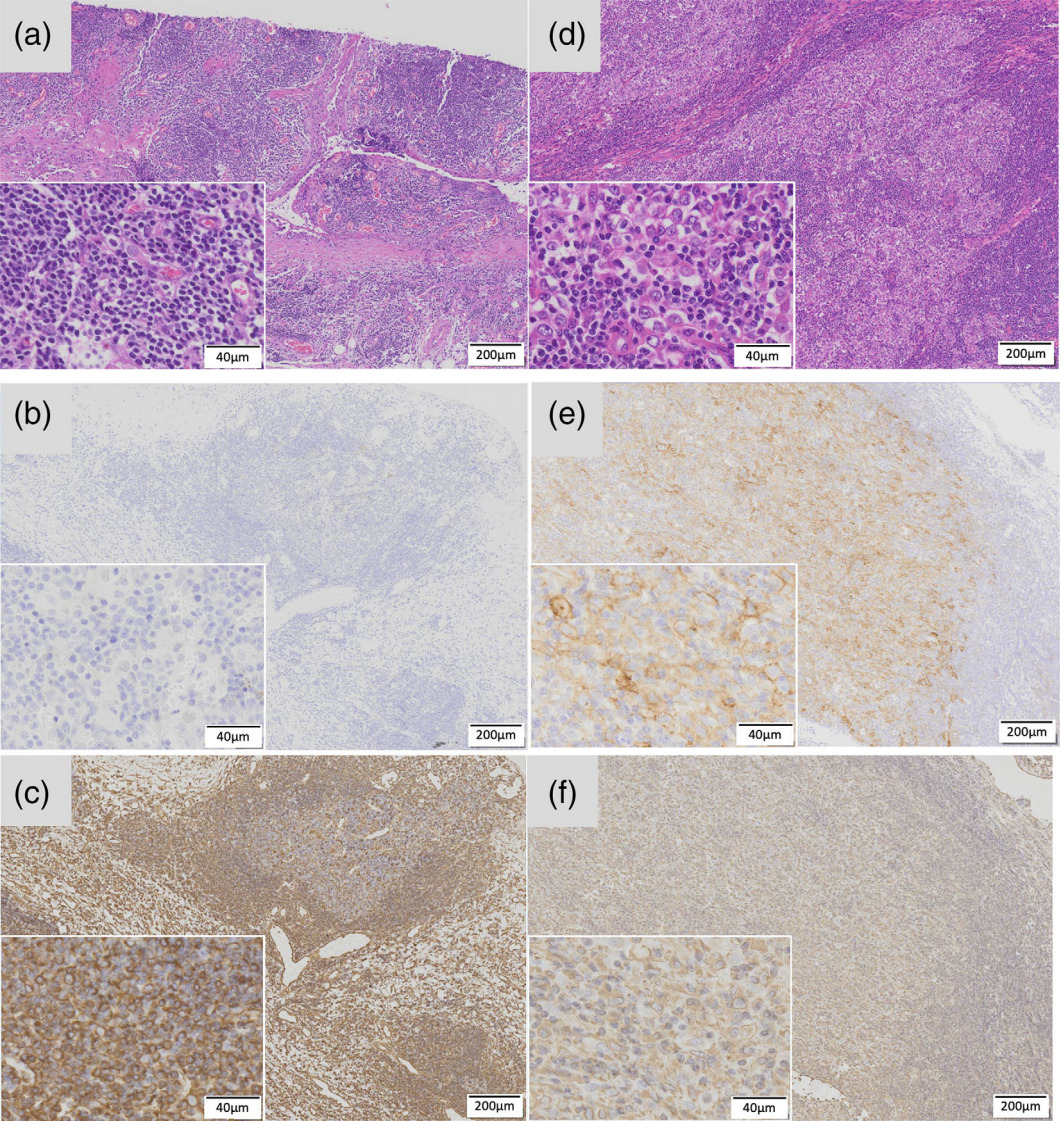
Immunohistochemistry of malignant pulmonary mesothelioma. (a) and (d) show hematoxylin–eosin staining, (b) and (e) show programed cell death ligand‐1 (PD‐L1) immunohistochemistry, and (c) and (f) show vimentin immunohistochemistry. (a)–(c) and (d)–(f) are the same specimen. Both specimens are epithelioid‐type malignant pleural mesothelioma (MPM) and were treated with nivolumab as the second‐line chemotherapy after surgery. PD‐L1 was positive for (e) and the effect was observed after administration of nivolumab. Vimentin was highly positive for all eight MPM specimens. The lower left quarter of each image is an enlarged image of the tumor part of the specimen

Among cases with MPM, one (12.5%) had PD‐L1‐positive staining in the tumor cell. This particular case had highly positive PD‐L1 expression (Figure 2). After two courses of cisplatin (CDDP) plus pemetrexed (PEM) as postoperative chemotherapy, this case received nivolumab and subsequently achieved partial response, whereas another case with negative PD‐L1 expression received nivolumab as second‐line chemotherapy, but terminated due to progressive disease. A comparison between previously reported studies on PD‐L1 expression in MPM is provided on Table 3.

**TABLE 3:** A comparison between previously reported studies on PD‐L1 expression in MPM

	*n*	Sex (M/F)	Age (median)	PD‐L1 status (%)	Smoker (%)	Pleomorphic carcinoma	Spindle‐cell carcinoma	Giant cell carcinoma	Carcinosarcoma	Pulmonary blastoma	Stage I	Stage II	Stage III	Stage IV
Velcheti et al.^16^	13	10 /3	61	9 (69.2)	9 (69.2)	1	0	10	1	0	6	4	2	1
Pecuchet al.^38^	15	10 /5	64.2 (mean)	7 (46.7)	15 (100)	14	0	1	0	0	3	7	5	0
Vieira et al.^17^	75	59/16	61	38 (53)	72 (96)	69	69	69	9	0	15	33	23	4
Yang et al.^10^	148	117/31	62.5	54 (36.5)	104 (70.3)	Unknown	Unknown	Unknown	Unknown	Unknown	‐	69 (I, II)	71	8
Naito et al.^15^	35	25/10	65	21 (60)	29 (83)	35	0	0	0	0	10	14	10	1
Kim et al.^23^	41	33/8	65	37 (90.2)	29 (80.6)	41	0	0	0	0	12	15	12	2

### Survival

Follow‐up lasted for as long as medical records were available. Among those with PSC, two survived for approximately 10 years, whereas the remaining who had poor prognosis died within 5 years after some metastasis. No clear correlation was found between PD‐L1 expression and prognosis. Among those with MPM, four survived for more than 2 years, with the longest being 9 years. All four cases had stage I disease on diagnosis.

## DISCUSSION

Previous studies have shown high PD‐L1 positive rates ranging from 36.5% to 90.2% in PSC.^21^, ^22^, ^23^ Consistent with previous studies, the current study found that four patients (50%) presented positive for PD‐L1. A comparison of published studies regarding the immunohistochemistry of PD‐L1 in PSC is provided in Table 3. According to these studies, anti‐PD‐1/PD‐L1 treatment may demonstrate potential therapeutic effects against PSCs, but the solid evidence has not yet been established.^24^, ^25^, ^26^, ^27^ Moreover, PD‐L1 tended to be positive in the sarcomatoid part of PSC, which was the vimentin‐positive area. Consistent with a previous study, the current study showed that three of the four cases with PD‐L1‐positive PSC showed positivity in the vimentin‐positive sarcomatoid area.^23^

Meanwhile, nivolumab plus ipilimumab has been approved as a first‐in‐class regimen for unresectable MPM in the USA.^28^ However, studies regarding the association between PD‐L1 expression and dual ICI efficacy have been inconsistent.^18^, ^19^, ^20^ Our study showed that nivolumab administered as a second‐line treatment successfully treated our single sample of epithelioid type MPM that showed positive expression of PD‐L1 (Figure 2c,d), inconsistent with previous studies. Therefore, only two patients underwent ICI as a second‐line treatment at our institution and one of them showed therapeutic effect and positive for PD‐L1; performing ICI in other cases could have possibly prolonged their prognosis as well. Nonepithelioid cell types have been reported to have a poor prognosis. Consistent with previous studies, our study showed that our single case presenting with biphasic histology died 3 months after surgery.^1^, ^2^ According to the phase 3 trial of Baas et al., the effect of the dual ICI can be expected even in the nonepithelioid type with poor prognosis. It can be said that the nonepithelioid type, which consists of sarcomatoid and biphasic types, has interstitial properties. In addition, it has been reported that vimentin, which is a medium‐diameter filament peculiar to stromal cells, is positive in MPM in 57–71% of cases.^29^, ^30^, ^31^ Since all eight cases in this study were positive for vimentin, it is considered that all of them had interstitial properties regardless of histologic subtypes. In this study, the patient who only obtained partial response by the second‐line treatment of nivolumab also had an epithelial type of MPM, but we speculate that the partial response may be due to positivity of vimentin and interstitial properties. While a previous study showed that stage III and higher disease stages accounted for approximately 70%, only one case in the current study was stage III, whereas the other cases were stage I. Some cases can achieve long‐term survival of 7 or 9 years, which has formed the basis for supporting the effectiveness of early detection and surgery.^1^, ^2^

Our study showed that there is no relationship between PD‐L1 positivity and survival in PSC, while ICI chemotherapy may improve the prognosis of PD‐L1‐positive cases in MPM, since the only case which was positive for PD‐L1 was improved by ICI chemotherapy. In both PSC and MPM, early clinical stage tended to result in better survival.

Thoracic mesenchymal malignant tumors such as MPM and PSC tend to have “mesenchymal” characteristics, such as having a sarcomatoid morphology or having vimentin, a medium‐diameter filament peculiar to stromal cells, in the cytoskeleton. In this study, it was shown that having this “interstitial” property may be related to the expression of PD‐L1 and the effect of ICI. Although MPM has already shown the effectiveness of ICI containing nivolumab, evidence of anti‐PD‐1/PD‐L1 inhibitor has not yet been established in PSC. Considering these reasons, it can be expected that ICI containing anti‐PD‐1/PD‐L1 inhibitor also will be effective in PSC if it has interstitial properties similar to MPM. For the treatment of PSC and MPM, combination therapy of multiple drugs, including ICI, has been utilized. Recently, several groups have reported on cluster of differentiation 47 (CD‐47), a protein highly expressed in small‐cell lung carcinoma that helps tumors to avoid immunity.^21^, ^32^, ^33^, ^34^ Yang et al. suggested that PD‐L1/CD47 co‐expression was an independent prognostic factor and may serve as a predictive biomarker for combined dual‐targeting immunotherapy for PSC. Baas et al. stated that combination therapy of anti‐PD‐L1 antibody and anti‐CTLA4 antibody significantly extended the overall survival of MPM in a phase 3 trial. Moreover, some cases of anti‐angiogenic therapy followed by nivolumab plus ipilimumab showed durable survival benefit in that trial. Studies on NSCLC and case reports on PSC have already reported regarding combination treatment involving ICI and anti‐angiogenic agents.^35^, ^36^ Given the various pathological features of PSC and MPM, multiple specific oncogene mutations may seemingly be involved. Several studies using next‐generation sequences are currently attempting to identity PSC‐ or MPM‐specific gene mutations.^37^, ^38^, ^39^ Thus, continued research on target gene mutations of PSCs through gene analysis like next‐generation sequencing seems necessary.

The current study has some limitations worth noting. First, given the single‐center, retrospective nature of this study, certain biases might exist. Second, our sample size was small and may be inadequate to obtain reliable results. Thus, further studies with a larger cohort are necessary to confirm our results.

## CONCLUSIONS

After evaluating eight cases each of PSC and MPM at our hospital, the current study found that SCs had higher PD‐L1 expression compared to NSCLC and that PD‐L1 expression tended to occur in the sarcomatoid compartment of both PSC and MPM. Moreover, ICI can prolong the prognosis of both tumors to some extent. Furthermore, combined ICI therapy containing anti‐PD‐L1 antibody and other immunotherapies or anti‐angiogenesis inhibitors is expected to further improve prognosis. To establish new combination therapies, other oncogene mutations need to be identified.

## CONFLICT OF INTEREST

All authors have declared no competing interests for this study.
