# Correction to “Thoracic mesenchymal malignant tumors and programed cell death ligand‐1 status: Clinicopathologic and prognostic analysis of eight pulmonary sarcomatoid carcinomas and eight malignant mesotheliomas”

**DOI:** 10.1111/1759-7714.15142

**Published:** 2023-10-25

Otsubo K, Sakai H, Kimura H, Miyazawa T, Marushima H, Kojima K, et al. Thoracic mesenchymal malignant tumors and programed cell death ligand‐1 status: Clinicopathologic and prognostic analysis of eight pulmonary sarcomatoid carcinomas and eight malignant mesotheliomas. Thorac Cancer. 2021;12:3169–76. https://doi.org/10.1111/1759-7714.14177

In Methods section, under the heading “Patients,” the Accession number should read as “4985” instead of “1461”. The correct sentence is shown below:

This study was approved by the institutional review board of the St. Marianna University School of Medicine in Kanagawa, Japan (Accession No. 4985).

We apologize for this error.

